# Effect of Sanitation Interventions on Health Outcomes: A Systematic Review of Cluster-Randomized Controlled Trials in Rural Communities of Low- and Middle-Income Countries

**DOI:** 10.3390/ijerph18168313

**Published:** 2021-08-05

**Authors:** Artwell Kanda, Esper Jacobeth Ncube, Kuku Voyi

**Affiliations:** Faculty of Health Sciences, School of Health Systems and Public Health, University of Pretoria, Private Bag 323, Pretoria 0007, South Africa; encube@randwater.co.za (E.J.N.); kuku.voyi@up.ac.za (K.V.)

**Keywords:** basic sanitation, health outcome, low- and middle-income countries, randomized controlled trial

## Abstract

A systematic review of published literature (2000–2019) evaluating the impact of sanitation interventions on the prevalence of disease, parasite infestation, and/or child growth using randomized controlled trials (RCTs) was done according to the PRISMA checklist. Earlier reviews indicated mixed evidence citing relatively poor quality evidence from mixed designs. Public health policy and practice appear to rely on evidence from RCTs. Records were searched in six electronic databases. The methodological quality of RCTs was assessed using the Cochrane collaboration risk of bias tool. Fifteen records (2.0%) were included for review. Impact trials were done in rural communities of African and Asian countries. The significant effect of sanitation-focus interventions was found in one trial for the prevalence of childhood diarrhea (14.3%), three trials for parasite infestation (37.5%), and two trials (25.0%) for child growth. Results indicate mixed quality evidence from RCT designs. Evidence is limited and suggestive of the impact of sanitation on parasite infestation and child growth. Further rigorous sanitation intervention trials under varying settings are needed to show what really works and under what settings. Future work may explore sanitation behavior change strategies and latrine options to address the challenges of poor latrine use under high sanitation coverage.

## 1. Introduction

Sanitation intervention impact research informs public health policy and practice. This could be particularly important for low- and middle-income countries (LMICs) where there is low access to basic sanitation [1], the burden of disease is borne [2], and sanitation remains a major health risk factor [1,3]. At the end of the millennium development goals era in 2015, about 32% of the global population (2.4 billion) still lacked access to improved sanitation, 70% living in rural areas [4]. Rural sanitation has become a priority task area. Several studies point to the significant reductions in the prevalence of diarrhea and enteric parasites and child growth with improvements in water, sanitation, and hygiene (WASH). However, it remains not very clear which specific interventions offer the most benefits and under what settings. Evidence from various research designs is mixed and too inconclusive to inform sanitation policy and practice.

A brief review of the literature highlights what is known. A review of 39 studies (1985–2003) by Fewtrell and Colford [5], which evaluated the effect of WASH on diarrhea, found that only one study was on sanitation alone. Wolf et al. [6] identified 11 studies of mixed designs that evaluated the effect of sanitation on health from 1970–2013. Most interventions were implemented as combined WASH. However, the specific effect of a single-focus intervention (e.g., sanitation) cannot be disaggregated from those of the commonly implemented combined WASH interventions [7]. A systematic review of the literature up to September 2016 on the effect of WASH on childhood diarrhea [8] identified one study specifically on sanitation alone. The study had no significant effect on childhood diarrhea. Overall estimates showed a 25% mean diarrheal risk reduction compared to a control group without intervention in a review of studies from 1970 to 2016 [9]. However, authors noted limited evidence.

Sanitation improvements were found to reduce the prevalence of soil-transmitted helminth (STH) infection in a systematic review and meta-analysis [10]. The authors reported that most of the evidence was from cross-sectional studies. Further, no randomized controlled trials (RCTs) were identified in their review. A similar review of 94 records up to October 2013 identified only five RCTs among the studies on sanitation [11]. Access to sanitation was found to be associated with a decreased likelihood of infection with any STH (odds ratio (OR) 0.66, 95% CI: 0.57–0.76), but not with hookworm. As in other reviews, data were considered to be of low quality due to there being many observational studies. A systematic review and meta-analysis that evaluated 54 studies up to June 2014 found that the availability or use of a sanitation facility was associated with lower odds of infection with *Entamoeba histolytica* or *Entomoeba dispar* (OR 0.5, 95% CI: 0.42–0.74) and *Giardia intestinalis* (0.64, 0.51–0.81) [12]. Only two of the studies were RCTs, the rest were observational. This is in agreement with similar work where mixed evidence was attributed to observational studies [13].

Demographic health survey data from 34 countries showed that the disposal practice of child feces in an improved toilet was associated with a 0.12 increase in height-for-age Z-score (HAZ; 95% CI: 0.10–0.15) [14]. In a systematic review of the effect of sanitation on childhood (<18 years) growth in LMICs, anthropometric measurements suggested little or no evidence [15]. Finally, a systematic review by Freeman et al. [16], which added 64 more studies than in earlier similar work up to December 2015, confirmed positive impacts of sanitation on health outcomes (diarrhea, active trachoma, some STHs, and height-for-age). The authors reported that the overall evidence was generally of poor quality with high heterogeneity.

The use of RCTs to determine the effect of sanitation interventions on health outcomes in rural communities is currently receiving great research attention. Earlier studies used mixed research designs, and they were mainly observational with few rigorous trials and reported mixed findings on the impact of sanitation alone on health outcomes with limited evidence. They lacked rigorous impact estimates due to limited study samples, robust designs, and credible control groups [17]. Despite potential methodological limitations, an RCT appears to be the design of choice in healthcare intervention impact research. The effect of an intervention in an RCT is tested by randomly allocating participants to sufficiently large and statistically balanced treatment and control groups [18]. A significant difference in the observed outcome is attributed to the intervention [19]. The current review includes some new large, rigorous RCTs that were not included in the latest review of various designs [16]. In the earlier review, which included 171 records up to the end of 2015, overall evidence suggested that sanitation is protective against diarrhea, active trachoma, some STH, and height-for-age.

The divergence of results and use of evidence from RCTs in sanitation interventions to inform public health policy and practice motivated this work. The review tries to answer the questions: Does new evidence from RCTs on sanitation interventions in rural communities of LMICs show consistent impacts on diarrhea, trachoma, child growth, and intestinal infection with earlier studies? What is the quality of the evidence? The work will be accomplished using evidence only from RCTs that evaluate the effect of sanitation interventions alone (not combined WASH) on selected health outcomes. This is perceived to contribute to the ongoing global research to understand the link between sanitation and health [20].

In this work, sanitation refers to having access to and using facilities and services to manage human excreta [20]. Sanitation intervention is considered to simply mean an increase in access to latrines. An outcome was taken to be a single end-of-intervention point with a linear causal-effect link to that intervention [21]. The health outcomes considered were the prevalence of disease or parasite infestation and the condition or state of body (growth) [22].

## 2. Materials and Methods

### 2.1. Search Strategy, Inclusion Criteria and Data Extraction

**Figure 1:**
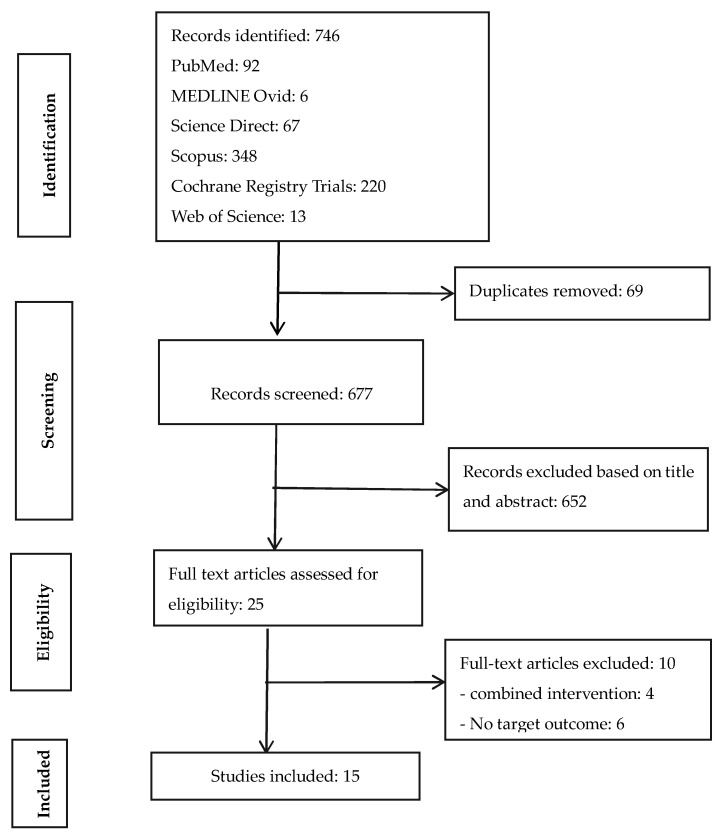
PRISMA flow chart of literature search.

The Preferred Reporting Items for Systematic Reviews and Meta-Analyses (PRISMA) checklist [23] was used to identify, screen, and include records for data extraction and analysis (Figure 1). A systematic review of published peer-reviewed literature was conducted between November 2019 and March 2020 for RCTs that evaluated the impact of sanitation interventions on disease/enteric parasite infestation, child growth, or their combinations as health outcomes indicators.

Electronic databases (Cochrane Library Trials (CENTRAL), MEDLINE—Ovid, PubMed, Science Direct, SCOPUS, and Web of Science) were searched for relevant records using appropriate search terms and filters (Table 1). The search stream was considered most appropriate after several ‘trial and error’ attempts. Analysis and synthesis of included records were done by two independent investigators.

The inclusion criteria considered peer-reviewed articles published in English from 1 January 2000 to 31 December 2019 that sought to evaluate the effect of sanitation interventions on health outcomes at rural the community level in LMICs based on RCTs. Interventions should have been be done at household (not school or hospital) level. Quasi-controlled trials, controlled before-and-after, and uncontrolled studies were excluded. Full-text screening identified the records for data extraction.

Data on the selected articles were extracted by two independent investigators. Upon discussion including a third investigator, discrepancies in the eligibility and extraction decisions were removed. A sheet with the characteristics of each study was prepared from the literature [18,24] and used to extract full reference, study area, intervention, participant characteristics, health outcomes, and key findings.

### 2.2. Assessment and Analysis of Included Studies

Qualitative assessment of included studies was done using five considerations: participants, intervention, health outcomes, bias assessment, and key findings derived from similar work [6,8]. The Cochrane collaboration risk of bias tool [25] was used to assess bias by two independent investigators who discussed with a third to reach consensus. Narrative synthesis was used for data analysis.

## 3. Results

### 3.1. Characteristics of Included studies

The literature search identified 746 studies from six electronic databases. Ten of the 25 full-text articles assessed for eligibility were excluded for not having a stand-alone sanitation intervention arm or the target health outcome indicators. Finally, 15 peer-reviewed publications from nine unique trails (different clinical registrations) were included (Figure 1). Studies were done in eight countries (five from Africa, three from Asia). About 93% of the studies were published from 2011 to 2019 and 86.7% had clinical registration numbers clearly indicated. Summaries of the 15 reviewed RCTs were categorized into the various characteristics suggested in the methodology and generally used in the literature (Table 2).

#### 3.1.1. Characteristics of Participants

Table 2 shows that the eligibility criteria for enrolment at household level included everyone greater than a given age limit, the presence of at least one child lower than a given age limit, the presence of a pregnant women in a given trimester, and the index child or non-index children within a given age limit at follow-up within the study area.

#### 3.1.2. Intervention, Adherence, Latrine Coverage, and Attrition at Follow-Up

All trials were cluster-randomized at village level, except for one at ward level [31]. In most cases, a trial profile was provided to show details of the intervention. Community participation in the interventions was mainly in the form of providing labor (such as pit digging and construction) and material for latrine construction (e.g., sand and bricks). Adherence (compliance) to intervention target behavior varied with trials and also during each trial. Baseline-endline sanitation coverage consisted of access to any (private/compound), improved, or functional latrine. Reasons for fall-out at follow-up were reported in 79% of the included studies shown on trial profiles. Follow-up times were from 0.5–2.5 years.

#### 3.1.3. Subsidies, Sanitation Demand, and Intention-to-Treat

Subsidies were provided for in cash or material, either to all participants or to households considered living below the poverty datum line. In some cases where the community-led total sanitation (CLTS) approach was used, material subsidies were provided in a government sanitation campaign. Participant demand for sanitation was triggered in the demand-side interventions, especially under CLTS or where its approaches were used. Without expressed demand for sanitation, even subsidized interventions (supply-side) e.g., [26,30,31,35] could not achieve total coverage and latrine use. Pit latrines with a plastic/concrete slab or pour flush system were the main technology options used in more than 60% of the interventions. However, different latrines built from local material (mainly unimproved) were constructed under CLTS programs. An intention-to-treat (ITT) was reportedly used to determine the difference between average target health outcomes across the sanitation intervention treatments and the control groups in 85% of the trials.

#### 3.1.4. Risk of Bias Assessment

**Figure 2:**
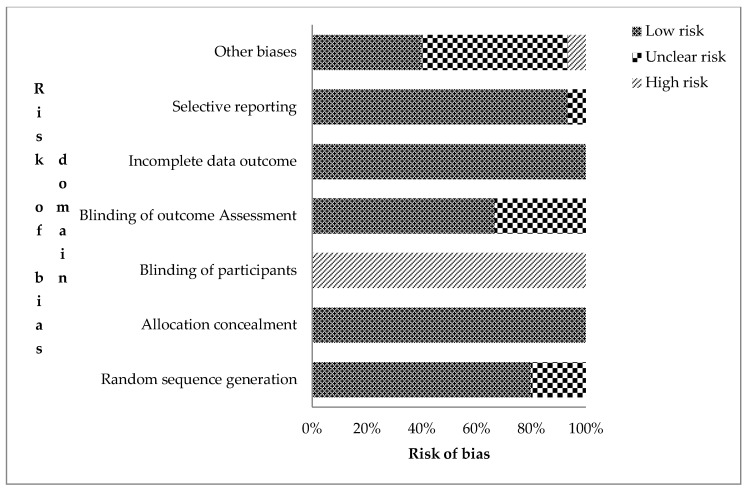
Risk assessment bias for the included cRCTs (*n* = 14) on the effect of sanitation on health outcomes in low- and middle-income countries (Authors’ judgement).

The authors’ risks of bias judgement for the included records are presented in the supplementary file Table S1. The overall assessment of risk of bias for the 15 RCTs is shown in Figure 2. Trials were judged based on the seven domains of the Cochrane collaboration bias assessment tool for undetected bias (low risk), detected bias (high risk), and uncertainty or lack of reported information (unclear risk of bias) [25]. Twelve RCTs (80.0%) were rated low risk of bias for sequence generation (selection bias). This means that the assigning of participants into treatment and control groups was randomized. In nine of them, a computer-generated randomization sequence allocation procedure was used by independent personnel. All studies were rated high risk for not blinding participants and field personnel. However, attempts were made to blind field personnel in some trials [3,30,34].

Ten trials (66.7%) were judged to have a low risk of detection bias as procedures of blinding outcome assessment were given. Loss to attrition, enrolment at follow-up, and intention-to-treat analysis were explained for all trials, resulting in low risk for attrition bias. Protocols and registered trials with predefined outcomes were available for 80% of the trials. Those without [17,26,35] were rated low risk of bias as the published reports included all pre-specified outcomes. Eleven trials, which relied on caregiver-reported diarrhea as a primary outcome, were judged unclear risk due to reporting bias.

### 3.2. Health Outcomes

Health outcomes (whether primary, secondary, or tertiary) upon which the effect of sanitation was assessed in the intervention, as indicated in the included studies, are shown in Table 3. Three main outcomes derived from the included studies were the prevalence of disease, parasite infestation, and child growth. Caregiver-reported that diarrhea and active trachoma were the two diseases considered. Parasite infestations were enteric helminths, protozoa, and other (*C. trachomatis*). The prevalence of disease was used in ten (66.7%), parasite infestation in nine (60.0%), and child growth (anthropometric measurements) in eight (53.3%) of the included trials. Only two RCTs (13.3%) considered all the three health outcomes under study in the sanitation impact interventions [3,29].

Results shown in Table 4 indicate that there was no significant effect on access to sanitation on the prevalence of disease, child diarrhea [3,17,29,30,31,34], or trachoma [26,27,28]. Reduction in the prevalence of trachoma in one study [26] was considered insignificant. Only one of the seven studies (14.3%) that investigated the impact of sanitation on the prevalence of child diarrhea showed positive results. The Bangladesh trial [33] showed that a 7-day diarrhea prevalence was lower among index children and children under 3 years at enrolment than the control in the sanitation intervention arm (PR 0.61, 95% CI 0.46–0.81).

Only two of the eight trials (25.0%) that assessed the impact of sanitation on child growth showed a positive effect [17,30]. The Mali CLTS trial showed that increased access to latrines improved child growth for the <2 years under conditions of high coverage with mostly unimproved latrines. CLTS children were taller (0.18 increase in HAZ, 95% CI 0.03–0.32; 2415 children) and less likely to be stunted (35% vs. 41%, PR 0.86, 95% CI: 0.74–1.0) than those from control villages [30]. The difference in mean weight-for-age z score (WAZ) for CLTS and control children was 0.09 (95% CI: −0.04 to 0.22) between groups. A similar trial setting of CLTS in Bhadrak, India, found an improvement in height-for-age z scores (0.37–0.52 and WAZ (0.26–0.31) standard deviations) relative to controls [17].

Three of the nine RCTs (33.3) that evaluated the effect of sanitation interventions on the prevalence of parasite infestation showed significant positive effects. The sanitation intervention on child enteric protozoan infections in rural Bangladesh [32] showed reduced prevalence of childhood *Giardia* infection in the treatment (26.5%, PR = 0.75 (0.64, 088)) than the control (35.5%) for 2.5-year old children. The CLTS intervention in rural Indonesia [35] showed a 48% reduction in roundworm infestation in treatment children relative to the control. Another trial in rural Bangladesh [36] showed that sanitation improvements reduced *T. trichiura* by 29% (PR = 0.71 (0.52, 0.98), Prevalence difference (PD) = −2.17 (−4.03 to 0.38)).

## 4. Discussion

We reviewed 14 RCTs that evaluated the impact of sanitation on pertinent health outcomes (diarrhea, trachoma, and child growth and parasite infection) from 2000 to 2019 in rural communities of LMICs. This was to find out whether evidence from RCTs was consistent with earlier findings from mixed design reviews. The latest review [16] considered records up to 2015. The current review adds seven RCTs from then to 2019. A single trial showed a positive impact of sanitation on childhood diarrhea. This could be a chance finding. Improved sanitation services had mixed findings on child growth (height and weight) and parasite infestation.

Participant enrolment based on households with pregnant women in some of the included trials could involve a small proportion of local residents [33]. Further, purposively selected countries or states where government interventions were in progress could limit researcher control of the intervention [39]. WASH interventions are generally implemented in a participatory manner with communities for sustainability and latrine use concerns [40]. Adherence to sanitation behavior helps reduce exposure [34]. This should not be assumed as it can distort interpretation of evidence by ignoring access to the sanitation technology-exposure link [41].

High coverage, access to, and functionality of a latrine may not result in its effective use as multi-level factors influence the adoption of a sanitation technology option [42]. This could explain the existence of open defecation and unused latrines in CLTS interventions with increased coverage [17,35]. Garn et al. [42] revealed a modest impact of sanitation interventions on increasing coverage and use. Higher latrine use was associated with type than low use in poor conditions. However, Massa et al. [43] considered effective latrine use as more important than its state. Finally, increased coverage remains important as there would be no point of measuring the health effects of a sanitation intervention without a ‘reasonable’ increase in coverage [44]. Post-intervention follow-up time influences the adoption of latrines [45]. Long periods introduce administrative treatment challenges such as non-adherence, contamination, and loss to follow-up [46], while short times may introduce the Hawthorne effect. Future work may evaluate optimum follow-up times where expected behavior is observed under given contextual settings.

Risk assessment data showed low risk of bias for most dimensions except for the blinding of participants. Central computer randomization was assumed to sufficiently conceal intervention allocations (low risk) [47]. Participants and caregivers are difficult to blind in community-based interventions [48], particularly where visible hardware, such as a latrine, is involved. Further, certification and signage declaring open defecation-free zones in CLTS interventions are visible to all. Self-reported diarrhea could be influenced by this, but intestinal infections and height-for-age were measured precisely to mitigate this concern. Different masked personnel in participant recruitment, data collection, and laboratory analyses strengthens the causal implications of the sanitation intervention on health outcomes [19] and therefore removes performance bias. Participant-reported information potentially suffers from response bias [49]. However, the potential effect to outcome assessment could not be ascertained, thus there was an unclear risk of bias. Clinical registration numbers were used as a non-statistical approach to evaluate publication bias [50].

Current health practice appears to rely mainly on evidence from RCTs. Earlier reviews indicated that few studies of mixed designs evaluated the effect of sanitation on diarrhea and child growth [6,8]. Improved coverage and reduced open defecation were reported but with limited significant effect to the prevalence of diarrhea and trachoma. Recommendations were the need to achieve total coverage to achieve health gains. However, this may need further enquiry if sustained use is not considered. The provision of sanitation services has to go beyond having access to a facility (hardware) to increase coverage. A latrine has to be accepted and effectively and consistently used, starting at household to the community level in rural areas. Various factors that influence latrine uptake have to be considered, including user preference. Sanitation technologies that include collection, containment, treatment, and disposal of fecal matter at once on site may help reduce multiple human exposure routes through the sanitation service chain. This is because health benefits may be realized by considering the whole sanitation service chain from the interface to disposal, including hygiene. However, other factors influence the selection of such technologies. Hygiene becomes critical in reducing human exposure to fecal pathogens while consistently using latrines. Efforts to end open defecation should also discourage having unimproved latrines at home and unhygienic latrine use.

A consistent lack of significant effect of improved sanitation to the prevalence of diarrhea from RCTs appears contrary to earlier reviews [51] with few such trials. The literature suggests that observational studies were considered to have poor quality evidence as they lack credible control groups, robust research designs, and large samples [17], and they are generally considered subject to bias [52] and cannot demonstrate causality. Observational studies cannot account for spillovers, a very significant issue in sanitation intervention research. Spillovers are intervention benefits enjoyed by those not directly participating. If spillovers are not accounted for, then the full public health benefits are underestimated.

The systematic review was aimed at assessing the current knowledge on whether there is consistent evidence from RCTs on the effect of sanitation on health outcomes by adding on new trials and identifying methodological limitations that could inform and improve future work. It was done without meta-analysis owing to the few trials available. Limitations to the current review included the use of only three out of the other possible health outcomes [53]. Further, the exclusion of records from grey literature and those not reported in the English language, and different combinations of literature search terms used could have left out other studies for inclusion in the review. Exclusion of interventions from grey literature may increase the risk of publication bias and threaten the validity of findings [54]. However, bias would most likely favor positive results (bias estimates upwards) whereas much of the findings, especially for the prevalence of diarrhea, show a lack of impact, so bias would not change the qualitative conclusion. The inclusion of multiple publications from the same intervention (with different health outcomes) under the same settings may overestimate the use of RCTs in sanitation interventions. The assessment of bias risk was done using a subjective instrument (Cochrane risk assessment tool), although two independent investigators were involved

## 5. Conclusions

Reviewed trials were done under varying settings such as socio-cultural, environmental, political, sanitation systems, approaches, and follow-up times. However, all RCTs that assessed the impact of sanitation on the prevalence of diarrhea, except one, consistently showed a lack of significant effect despite varying settings and increase in coverage. This may point to the need for combined WASH programming to respond to multiple environmental exposure pathways. However, access to sanitation remains a human right and has other associated benefits. The observed positive impact of sanitation under a CLTS intervention where various technology designs (improved and unimproved) were used may highlight the importance of increased access to a latrine and effective use as opposed to technology design, an area still under scientific enquiry. The provision of targeted subsidy under CLTS approaches may highlight the importance of accessing latrines by the poor. The review showed that a hybrid CLTS approach with target subsidies was commonly used in the CLTS interventions opposed to the original tenets of the approach. This observation may require further field-based research evidence to inform sanitation practice. Based on the few sanitation-based RCTs available, there is limited and inconclusive evidence of the health benefits of sanitation-specific interventions on child growth and parasitic infestation. It may be difficult to inform sanitation policy and practice on WASH programming for intervention-specific approaches. Rigorous large-scale trials on similar health outcomes are still needed that achieve high sanitation coverage and latrine use. Sanitation behavior change strategies should address low latrine uptake under conditions of high coverage. Future work may consider the extent to which a sanitation intervention facilitates reduction in the prevalence of parasite infestation and improves child growth in view of the multiple environmental exposure pathways and the optimal time frame when the health outcome is measured.

## Figures and Tables

**Table 1:** Literature search terms.

Database	Search String	Applied Filters
Cochrane Library-Trials	Advanced search: (WASH sanitation randomized controlled trial)	2000–2019 English
MEDLINE Ovid	Advanced search: ((((effect OR impact) AND (sanitation OR WASH) AND interventions AND health AND outcomes) OR (disease OR diarrhea OR child growth)) AND (randomized AND controlled AND trial) AND (low AND middle AND income AND country))	2000–2019 Article Full text journals
PubMed	Advanced search: (sanitation interventions health outcomes randomized controlled trials)	Full text 2000–2019 Randomized controlled trial
Science Direct	Advanced search: (effect sanitation interventions health outcomes randomized controlled trials low- and middle-income countries)	Research article 2000–2019
SCOPUS	Advanced search: (effect OR impact AND sanitation OR WASH AND interventions AND health AND outcomes OR diarrhea OR child AND growth AND randomized AND controlled AND trial AND low- AND middle- AND income AND country)	2000–2019 Article English
Web of Science	Advanced search: TS = (effect AND sanitation AND interventions AND diarrhea AND child AND growth AND randomized AND controlled AND trials)	2000–2019 English

**Table 2:** Summaries of random controlled trials included for the review.

Reference	Country/Continent of Trial/Trial Registration	% Access to Basic Sanitation	Sanitation Intervention Group	Intervention Duration (Years)	Sanitation Technology Option(s)	Sanitation Demand	Exposure Pathway(s) Based on the Study	Intervention Subsidy	Reasons for Loss to Follow-Up
Emerson et al. [26]	Gambia (Africa) -	3.5	2230 participants in 7 clusters	2	Non-ventilated pit latrine	Not specified	Vector Contact	Government subsidized	travelled, death, declined
Gebre et al. [27]	Ethiopia (Africa) NCT00322972	-	14,189 persons in 12 Subkebeles	2.16	Pit latrine with concrete slab	Not specified	Vector Contact	Government subsidized	-
Stoller et al. [28]	Ethiopia (Africa) NCT00322972	-	14,289 people in 12 Subkebeles	2	Simple pit latrine	Not specified	Vector	Material subsidy	-
Clasen et al. [3]	India (Asia) NCT01214785	9 (any type)	10,014 individuals, including 1919 chn <5 in; 50 villages	3.58	Pour flush	Not specified	Water, Contact, food	Government subsidized	Death, absent, family dropout
Patil et al. [29]	India (Asia) NCT01465204	13.64	1683 chn < 5976 households in 40 villages	Not clear	Various	Not specified	Water, food contact	Government subsidized for national TSC	-
Dickinson et al. [17]	India (Asia) -	25 (owned)	1050 households, 1256 chn <5, 40 villages	0.42	Several under CLTS	CLTS triggering	Water	Government subsidized	-
Pickering et al. [30]	Mali (Africa) NCT01900912	22 (control)	2365 households, 3508 chn <5, 60 villages	Not clear	Several under CLTS	CLTS triggering	Water	-	-
Briceño et al. [31]	Tanzania (Africa) NCT01465204	49.7	86 villages in 44 wards	2.3	Several under CLTS	CLTS triggering	Water, food contact	-	-
Lin et al. [32]	Bangladesh (Asia) NCT01590095	53 (owned)	696 compounds in 90 clusters	1	Double pit Latrine with water seal	Not specified	Water, contact	Material subsidy	Moved, death, withdrew, no live birth, absent
Luby et al. [33]	Bangladesh (Asia) NCC01590095	54 (owned)	696 compounds in 90 clusters	1	Double pit Latrine with water seal	Not specified	Water, contact food	Material subsidy	Moved, no live Birth, absent, refused,
Null et al. [34]	Kenya (Africa) NCT01704105	16	892 households 77 clusters	1.5	‘Improved latrines’	Not specified	Water, food contact	Material Subsidy,	Absent, died refused, no live birth,
Cameron et al. [35]	Indonesia (Asia) -	-	80 villages	-	Several CLTS campaign	CLTS triggering	Contact, food water	-	-
Ercumen et al. [36]	Bangladesh (Asia) NCT01590095	53.4 (owned)	696 women in 90 clusters, 1030 children	1	Concrete-lined double pit latrine (seal)	Not specified	Contact water	Provision of upgraded latrines	Moved, death, absent, no live birth, withdrew
Pickering et al. [37]	Kenya (Africa) NCT01704105	15.7	892 households in 77 clusters	1.5	Not specified	Not specified	Water, food contact	New latrines and upgrading existing ones	Absent, death, refused, no live birth
Stainbaum et al. [38]	Kenya (Africa) NCT01704105	15.7	892 households 77 clusters	1.5	‘Improved latrines’	Not specified	Water, food contact	Material subsidy	Absent, no live Birth, refused, death
**Reference**	**Time When** **Post-Intervention Follow-up Was Done (In Years)**	**Enrolment Criteria**	**Intervention** **Adherence (%)**	**Health** **Outcome**	**Study Limitations**			**Key Findings**	
Emerson et al. [26]	0.5	Clusters randomly recruited in sets of three	First 0.5 years: 98%	Disease	Study done in low prevalence area. Fly catching without release induces catcher bias (unblinded)			Access to basic sanitation reduced fly eye contact Insignificant reduction in prevalence of trachoma in sanitation intervention	
Gebre et al. [27]	2.16	Subkebekes randomly selected	61.5	Disease	No masking, insufficient sample size, no hygiene education			No effect of latrine construction on mortality (under 5 year old children).	
Stoller et al. [28]	1 and 2	Subkebekes Randomly selected	67.2	Disease, Parasite	Flies not only transmission route, sanitation control varies in space and time,			Latrine construction offered no protection to prevalence of trachoma	
Clasen et al. [3]	1.5	Household with child <4 years or pregnant woman	36	Disease, Growth, Parasite	Short follow-up period 1.5 year Self- and care-giver reported bias			No reduced exposure, prevention to diarrhea and STHs or child effect on malnutrition.	
Patil et al. [29]	1.75	Villages randomly selected	59	Disease, Growth, Parasite	Short-term follow-up, contamination in the control group and self-reported outcomes			Increased coverage (19%), reduced open defecation 10%) but no improvements on diseases and child growth	
Dickinson et al. [17]	0.42	Household with child <5 years	-	Disease, Growth	Study under-powered to statistically detect precise effects on diarrhea,			No statistically precise reductions in diarrhea, but increased anthropometric measurements of children <5 years of age	
Pickering et al. [30]	1.5	Household with at least a child <10 years old	-	Disease, Growth	Self-reported measure, one follow-up in dry season, no universal access			No reduced diarrhea prevalence, increased child growth (<2) reduced open defecation and stunting (<5). Future work: Sanitation and height	
Briceño et al. [31]	1	Households with a child <5	-	Disease, Growth	No pre-intervention baseline characteristics, small changes in intermediate outcomes due to isolated interventions			Increased access (49.7–64.8%), reduced open defecation but the final effects of sanitation on child health were absent	
Lin et al. [32]	2.5	Pregnant women, Chn ages 18–27 months	85–87	Parasite	*Giardia* genotype not determined, unknown protozoan infection status after intervention initiation but determined before 2 years.			Sanitation intervention reduced Childhood *Giardia* infections (9%)	
Luby et al. [33]	1 and 2	Pregnant women, Index chn	‘high’	Disease, Growth	Caregiver-reported primary outcomes. Intervention in one socio-ecological zone in times of low prevalence of diarrhea			Sanitation intervention had no effect on child linear growth at year 2 but reduced the diarrhea prevalence (3.5%) than in the control (5.7%).	
Null et al. [34]	1 and 2	Pregnant women, other requirements	>70: year 1, <25: year 2	Disease, Growth	No observable indicators of actual behavior, compound and not community-level, focus on human feces not animal			Sanitation had no effect on diarrhea prevalence and child growth.	
Cameron et al. [35]	2	Household with child <5 years	‘low’	Parasite, Growth	Partial compliance to treatment as 13.8% of the control was exposed to treatment			Associated decrease in roundworm infestations but no improvements in child growth.	
Ercumen et al. [36]	2.5	Pregnant women in 1st or 2nd trimester, Index chn	54	Parasite	Poor instrumental sensitivity, only relative statistical power to detect relatively large effects, short follow-up for *A. lumbricoides*			Sanitation reduced *T. trichiura* (29%), had borderline effects on hookworm and no effect on *A. lumbricoides*.	
Pickering et al. [37]	2	Village with at least 6 pregnant women Index chn	Year 1: 89–90 Year 2: 79–82	Parasite	Imperfect uptake of targeted behaviour, limited power to detect effects on rareparasite infections			Sanitation had no effect on prevalence of *Ascaris* infection, and no reduction in *Giardia*	
Steinbaum et al. [38]	2	Village with pregnant women, Index chn	Year 1: 89–90 Year 2: 79–82	Parasite	No precise measures of compound defecation practices Soil analysis method only optimized for *Ascaris*, not *Trichris* or hookworm eggs			Sanitation had no effect on presence of eggs of total STH, *Ascaris* or *Trichuris*	

Chn: children; CLTS: Community-Led Total Sanitation; TSC–Total Sanitation campaign.

**Table 3:** Main health outcomes upon which the effect of sanitation was assessed in the intervention as indicated in the included studies.

Reference	Presence of Disease		Parasite Infestation			Child Growth	Main Indicator (s) for the Outcome					Total Outcomes
	Diarrhoea	Trachoma	Protozoan	Helminthic	Other	Anthropometric	Prevalence	Mortality	Height	Weight	Other	
Emerson et al. [26]		✓					✓					1
Gebre et al. [27]		✓			✓			✓				2
Stoller et al. [28]		✓			✓		✓					2
Clasen et al. [3]	✓			✓		✓	✓		✓	✓	✓	3
Patil et al. [29]	✓			✓		✓	✓		✓	✓	✓	3
Dickinson et al. [17]	✓					✓	✓		✓	✓	✓	2
Pickering et al. [30]	✓					✓	✓		✓	✓		2
Briceño et al. [31]	✓					✓	✓		✓	✓		2
Lin A et al. [32]			✓				✓					1
Luby et al. [33]	✓					✓	✓		✓	✓	✓	2
Null et al. [34]	✓					✓	✓		✓	✓	✓	2
Cameron et al. [35]				✓		✓	✓		✓	✓		2
Ercumen et al. [36]				✓			✓					1
Pickering et al. [37] Steinbaum et al. [38]			✓	✓ ✓			✓ ✓					1 1

**Table 4:** Summary of results showing the effect of sanitation interventions on disease, parasite infestation and child growth.

Health Outcome		Significant Effect of Sanitation Shown by Randomised Controlled Trial														
		1	2	3	4	5	6	7	8	9	10	11	12	13	14	15
Disease	Active trachoma	✕	✕	✕												
	Reported diarrhoea				✕	✕	✕	✕	✕		✓	✕				
Parasite infection	protozoa									✓					✕	
	Enteric helminths				✕								✓	✓	✕	✕
	Other		✕	✕												
Child growth (anthropometric)	Weight				✕	✕	✓	✓	✕		✕	✕	✕			
	Height				✕	✕	✓	✓	✕		✕	✕	✕			
	Other measure				✕	✕	✓				✕	✕				

✓—Significant effect; ✕—No significant effect; 1—Emerson et al. [26]; 2—Gebre et al. [27]; 3—Stoller et al. [28]; 4—Clasen et al. [3]; 5—Patil et al. [29]; 6—Dickinson et al. [17]; 7—Pickering et al. [30]; 8—Briceño et al. [31]; 9—Lin et al. [32]; 10—Luby et al. [33]; 11—Null et al. [34]; 12—Cameron et al. [35]; 13—Ercumen et al. [36]; 14—Pickering et al. [37]; 15—Steinbaum et al. [38].

## Data Availability

No new data were created or analyzed in this study. Data sharing is not applicable to this article.

## Supplementary Materials

The following are available online at https://www.mdpi.com/article/10.3390/ijerph18168313/s1, Table S1: Assessment of risk of bias for 15 RCTs used to determine the impact of sanitation on health outcomes (Adapted from The Cochrane Collaboration’s tool for assessing risk of bias).
