# Supplementary material for: Effect of Sanitation Interventions on Health Outcomes: A Systematic Review of Cluster-Randomized Controlled Trials in Rural Communities of Low- and Middle-Income Countries
Source: Int J Environ Res Public Health. 2021 Aug 5;18(16):8313. doi: 10.3390/ijerph18168313 (PMC8392128; doi:10.3390/ijerph18168313)
Supplement: Supplementary file 1 [file ijerph-18-08313-s001.zip › ijerph-1287862-supplementary.pdf]

**Supplementary Material**. Assessment of risk of bias for 15 RCTs used to determine the impact of sanitation on health outcomes (Adapted from The Cochrane Collaboration's tool for assessing risk of bias)

| Reference | Domain | Reviewers' judgement | Support for judgement |
|---|---|---|---|
| Emerson et al. 2004 [26] | Random sequence generation | Low risk | Clusters recruited in sets of 3, randomly assigned to treatments/control by drawing from a hat |
| | Allocation concealment | Low risk | Clusters at community level, their recruitment unlikely to influence participant recruitment |
| | Blinding of participants & personnel | High risk | One latrine was allocated per household. Difficult to blind when latrine hardware was provided |
| | Blinding of outcome assessment | Low risk | All participants screened, both eyes inspected, single photograph taken and blinded clinicians |
| | Incomplete outcome data | Low risk | All clusters participated and were visited 3 times, participants lost to follow-up did not differ |
| | Selective reporting | Low risk | Active trachoma, primary outcome was reported |
| | Other biases | Low risk | No clusters were lost at follow up, proportions with active trachoma statistically not different |
| Gebre et al. 2011 [27] | Random sequence generation | Low risk | Randomization sequence was computer-generated in MS Excel |
| | Allocation concealment | Low risk | Concealment mentioned without details but we judged central randomisation to sufficiently conceal intervention allocations |
| | Blinding of participants & personnel | High risk | Subkebeles were not masked to the intervention |
| | Blinding of outcome assessment | Unclear risk | Separate authors did randomisation and participant enrolment. It is not clear who did the statistical analysis and mortality rate calculations (We), and its effect to outcome assessment |
| | Incomplete outcome data | Low risk | Census updated for deaths and migration, used in calculations. Repeat census to 24 sublevels |
| | Selective reporting | Low risk | Primary outcome 'age-specific all-cause mortality' pre-specified in the registered protocol reported |
| | Other biases | Low risk | Masked census auxiliary health workers to treatments and outcome. All sublevels were visited. |
| Stoller et al. 2011 [28] | Random sequence generation | Low risk | Randomization sequence was computer-generated in MS Excel for clinical comparisons |
| | Allocation concealment | Low risk | No detail, but we judged central randomisation to sufficiently conceal intervention allocations |
| | Blinding of participants & personnel | High risk | Intensification of an existing latrine programme, prior knowledge of treatment known |
| | Blinding of outcome assessment | Low risk | Swab samples from randomly selected participants at baseline and follow-up were pooled to detect ocular *C. trachomatis* by blinded laboratory personnel |
| | Incomplete outcome data | Low risk | Follow-up inclusions and exclusions described. Randomly sampled participants used in data collection. Intention-to treat analysis was done. |
| | Selective reporting | Low risk | Prevalence of *C. trachomatis* infection in children 0-9 years was reported as primary outcome |
| | Other biases | High risk | The two treatment groups were well balanced except for antibiotic coverage (cluster imbalance). on-going latrine construction programme (intervention contamination) |
| Clasen et al. 2014 [3] | Random sequence generation | Low risk | Cluster randomisation by computer-generated sequence |
| | Allocation concealment | Low risk | We judged central randomisation to sufficiently conceal intervention allocations |
| | Blinding of participants & personnel | High risk | Reported blinding of participants was not possible |
| | Blinding of outcome assessment | Low risk | Random assignment was by not being involved in data collection or intervention delivery |

| | | | |
|---|---|---|---|
| | Incomplete outcome data | Low risk | Village level clustering was statistically accounted for, adjustments at follow-up due to accounted-for attrition was given (baseline diarrhoea). Intention-to treat analysis was done. |
| | Selective reporting | Low risk | Primary outcome, 7-day diarrhoea prevalence was compared across treatments and control |
| | Other biases | Unclear risk | Care-giver self-reported data is prone to reporting bias. Its effect on the outcome is not clear |
| Patil et al. 2014 [29] | Random sequence generation | Low risk | Randomization took place by publicly picking lottery ticket to assign villages to treatments |
| | Allocation concealment | Low risk | We judged central randomisation to sufficiently conceal intervention allocations |
| | Blinding of participants & personnel | High risk | Programme implementers and researchers not blinded. Blinded interviewers could identify intervention villages during interviews of block officers or the village secretary. |
| | Blinding of outcome assessment | Unclear risk | Blinded interviewers could identify intervention villages during interviews. The effect to their data collection is not clear |
| | Incomplete outcome data | Low risk | No differential attrition by group, no missing data. Intention-to treat analysis was done. |
| | Selective reporting | Low risk | Study protocol's predefined outcomes were reported |
| | Other biases | Unclear risk | Care-giver self-reported data is prone to reporting bias. Its effect on the outcome is not clear |
| Dickinson et al. 2015 [17] | Random sequence generation | Low risk | Randomization took place by publicly picking slips from a bucket to assign villages to treatments |
| | Allocation concealment | Low risk | No detail but we judged central randomisation to sufficiently conceal intervention allocations |
| | Blinding of participants & personnel | High risk | Researchers/implementers were not blinded |
| | Blinding of outcome assessment | Unclear risk | Same authors did randomisation, participant enrolment and statistical analysis (We). Same enumerators collected data in base- and end line surveys. The effect to outcome assessment is not clear |
| | Incomplete outcome data | Low risk | Random subsamples within villages were used for data collection. Loss at follow-up was reported |
| | Selective reporting | Low risk | Pre-specified outcomes in the protocol were reported relative to the control. |
| | Other biases | Unclear risk | Care-giver self-reported diarrhoea data is prone to reporting bias. Its effect on the outcome is not clear |
| Pickering et al. 2015 [30] | Random sequence generation | Low risk | Computer-generated algorithm that randomly assigned villages to treatment and control groups |
| | Allocation concealment | Low risk | We judged central randomisation to sufficiently conceal intervention allocations |
| | Blinding of participants & personnel | High risk | Participants were not masked to treatment status |
| | Blinding of outcome assessment | Unclear risk | Although interviewees were blinded, they could infer status during interviews from presence of signage showing village certification of an open defecation free status w, with unknown effect |
| | Incomplete outcome data | Low risk | Attrition and re-inclusion at follow-up reported with numbers to balance groups. |
| | Selective reporting | Low risk | Registered study protocol with pre-specified outcomes were reported |
| | Other biases | Unclear risk | Care-giver self-reported data is prone to reporting bias. Its effect on the outcome is not clear |
| Briceño et al. 2017 [31] | Random sequence generation | Unclear risk | Factorial cluster-randomized control trial, 190 largest wards randomly sampled. Insufficient information about the sequence generation process to permit judgement of low or high risk. |
| | Allocation concealment | Low risk | Treatment groups knew their assignment, but not controls, unbeknown even to survey teams. However, knowing treatment (without controls) concealed knowledge of treatment comparisons |
| | Blinding of participants & personnel | High risk | Participants were not blinded |
| | Blinding of outcome assessment | Low risk | Survey firms were never provided information on treatment status of participating wards |
| | Incomplete outcome data | Low risk | No significant effect. Random subsamples within wards were used for data collection. |
| | Selective reporting | Low risk | Outcomes pre-defined in the protocol were reported |

| | | | |
|---|---|---|---|
| | Other biases | Unclear risk | Care-giver self-reported data is prone to reporting bias. Its effect on the outcome is not clear |
| Lin et al. 2018 [32] | Random sequence generation | Low risk | Random number generator used to randomise matched clusters to the double-sized control arm |
| | Allocation concealment | Low risk | We judged central randomisation to sufficiently conceal intervention allocations |
| | Blinding of participants & personnel | High risk | Study participants, intervention implementers, and outcome assessors were not masked because the interventions delivered visible hardware |
| | Blinding of outcome assessment | Low risk | Masked lab technician conducted analyses to detect protozoa infections. Two investigators conducted independent masked data processing and statistical analyses |
| | Incomplete outcome data | Low risk | Statistical analyses performed for loss and recovery to follow-up. Intention-to treat analysis was done. |
| | Selective reporting | Low risk | Registered trial protocol available. Pre-specified outcome (tertiary) of interest reported |
| | Other biases | Low risk | We judged the study to appear free of other sources of bias |
| Luby et al. 2018 [33] | Random sequence generation | Low risk | Clusters randomly allocated to treatment using a random number generator by a co-investigator |
| | Allocation concealment | Low risk | We judged central randomisation to sufficiently conceal intervention allocations |
| | Blinding of participants & personnel | High risk | Interventions included distinct visible components so neither participants nor data collectors were masked to intervention assignment |
| | Blinding of outcome assessment | Low risk | Independent, masked statistical analyses with the true group assignment variable replaced with a re-randomised uninformative assignment variable. |
| | Incomplete outcome data | Low risk | Loss to attrition with reasons and enrolment at follow-up were given. Intention-to treat analysis was done. |
| | Selective reporting | Low risk | Registered trial protocol available. Pre-specified outcome of interest reported |
| | Other biases | Unclear risk | Care-giver self-reported data is prone to reporting bias. Its effect on the outcome is not clear |
| Null et al. 2018 [34] | Random sequence generation | Low risk | Clusters were randomly allocated to treatment using a random number generator |
| | Allocation concealment | Low risk | We judged central randomisation to sufficiently conceal intervention allocations |
| | Blinding of participants & personnel | High risk | Cluster allocation was communicated directly to the field team, Participants were not blinded |
| | Blinding of outcome assessment | Low risk | Investigators remained blinded to treatment assignments. 2 blinded biostatisticians independently replicated the analyses following the pre-specified analysis plan |
| | Incomplete outcome data | Low risk | Monitoring data collected during unannounced visits to a random sample of at least 20% of participants in intervention groups at given time periods after the interventions began. Loss to attrition with reasons at follow-up was given. Intention-to treat analysis was done. |
| | Selective reporting | Low risk | Registered trial protocol available. Pre-specified outcome of interest reported |
| | Other biases | Unclear risk | Care-giver self-reported data is prone to reporting bias. Its effect on the outcome is not clear |
| Cameron et al. 2019 [35] | Random sequence generation | Unclear risk | Randomisation stratified at village and sub-village levels with comparison control groups described Insufficient information about the sequence generation process to permit judgement of low or high risk |
| | Allocation concealment | Low risk | We judged central randomisation to sufficiently conceal intervention allocations |
| | Blinding of participants & personnel | High risk | No detail was provided but we judged that interventions included distinct visible components (latrines), signage and certification of open defecation free zones, sanitation demand triggering sessions so neither participants nor data collectors were masked to intervention assignment |
| | Blinding of outcome assessment | Unclear risk | Insufficient information to permit reviewers' judgment of low or high risk |

| | | | |
|---|---|---|---|
| | Incomplete outcome data | Low risk | No imbalances in village characteristics. First and second rounds of visits were done with no details to attrition. We judged no incomplete outcome data due to stratified sampling for analysis |
| | Selective reporting | Low risk | Although we did not have the trial registration of the protocol, we judged low risk of bias as all outcomes reported |
| | Other biases | low risk | We judged the study to appear free of other sources of bias |
| Ercumen et al. 2019 [36] | Random sequence generation | Low risk | Off-site investigator used a random number generator to block-randomize clusters into study arms |
| | Allocation concealment | Low risk | We judged central randomisation to sufficiently conceal intervention allocations |
| | Blinding of participants & personnel | High risk | Participants and field staff were not blinded as interventions entailed distinct hardware |
| | Blinding of outcome assessment | Low risk | Blinded technicians enumerated STH outcomes, blinded analysts independently replicated data management and analysis |
| | Incomplete outcome data | Low risk | Loss to attrition with reasons, enrolment at follow-up were given and balanced in numbers across intervention groups. Intention-to treat analysis was done. |
| | Selective reporting | Low risk | Registered trial protocol available. Pre-specified primary outcome of interest reported |
| | Other biases | Low risk | We judged the study to appear free of other sources of bias |
| Pickering et al. 2019 [37] | Random sequence generation | Low risk | Independent investigator used a random number generator to randomly assign clusters |
| | Allocation concealment | Low risk | We judged central randomisation to sufficiently conceal intervention allocations |
| | Blinding of participants & personnel | High risk | Blinding of participants was not possible given the nature of the interventions |
| | Blinding of outcome assessment | Low risk | Blinded lab technicians analysed samples. 2 authors independently replicated the statistical analyses while blinded to intervention status. |
| | Incomplete outcome data | Low risk | Loss to attrition with reasons, enrolment at follow-up were given and balanced in numbers across intervention groups. No incomplete data |
| | Selective reporting | Low risk | Registered trial protocol available. Pre-specified outcome (tertiary) of interest reported |
| | Other biases | Low risk | We judged the study to appear free of other sources of bias |
| Steinbaum et al. 2019 [38] | Random sequence generation | Unclear risk | Authors referred to "We" in the methodology without indicating independent investigator to randomly assign clusters |
| | Allocation concealment | Low risk | We judged central randomisation to sufficiently conceal intervention allocations |
| | Blinding of participants & personnel | High risk | Blinding of participants was not possible as material subsidy was given for latrines. Blinding of the two laboratory technicians who did all sample analyses not clearly spelt status. |
| | Blinding of outcome assessment | Low risk | |
| | Incomplete outcome data | Low risk | Loss to attrition with reasons reported. No incomplete data |
| | Selective reporting | Unclear risk | Registered trial protocol available, but a not pre-specified outcome of interest reported. Effect unclear |
| | Other biases | Unclear risk | Use of not optimised method for other laboratory analyses may introduce 'other' unclear bias |
